# Quantification of pH tolerance levels among entomopathogenic nematodes

**DOI:** 10.21307/jofnem-2021-062

**Published:** 2021-07-08

**Authors:** Zanele Khathwayo, Tshimangadzo Ramakuwela, Justin Hatting, David I. Shapiro-Ilan, Nicolene Cochrane

**Affiliations:** 1 ARC-Small Grain, P/Bag X29, Bethlehem, 9700, South Africa; 2 USDA-ARS, Southeastern Fruit and Tree Nut Research Station, Byron, GA, 31008; 3 ARC-Biometry, Central Office, P.O. Box 1134, Pretoria, 0001, South Africa

**Keywords:** Ammonium-acetate, Citrate-phosphate, Entomopathogenic nematodes, pH levels, Survival

## Abstract

Soil pH affects the availability of nutrients, which impacts plant growth and development. Similarly, soil pH may also influence microorganisms in the soil, either beneficial or nonbeneficial. One such group of beneficial microorganisms is entomopathogenic nematodes (EPN), parasites of soil-inhabiting insects. Entomopathogenic nematodes have a number of attributes that make them good alternatives to chemical insecticides. The objective of this study was to investigate pH tolerance of 11 steinernematids and six heterorhabditids post exposure to different pH levels. Entomopathogenic nematode populations were exposed to varying pH levels (pH2 to pH11) made up from two different chemical solutions (ammonium-acetate and citrate-phosphate). Entomopathogenic nematode populations are expected to have varying tolerance to different pH levels. The highest infective juvenile survival was obtained from pH3 to pH10 in citrate-phosphate, where all populations displayed >50% survival. *Steinernema carpocapsae* populations had >90% survival at pH3 to pH11 in citrate-phosphate solutions. Overall, the steinernematids had a higher survival range in ammonium-acetate pH solutions compared with the heterorhabditids. Moreover, *Steinernema* spp., *S. carpocapsae* (ScCxrd, ScAll, and ScItalian) and *S. riobrave* showed consistently higher survival in both acidic and alkaline solutions, when compared to the other steinernematids, suggesting that they may be applied in both acidic and alkaline soils. These findings can be of use when selecting EPNs for biological control purposes in the two countries, respectively.

In agriculture, soil pH affects the availability of nutrients, which impacts plant growth and development. For plants, optimal pH ranges from 5.5 to 6.5 (Islam et al., 1980; Soti et al., 2015). In addition to its impact on plant growth and development, soil pH may also influence microorganisms in the soil through the so-called ‘oligodynamic effect’ (Shrestha et al., 2009); this phenomenon refers to the toxicity induced by metals (Cu, Zn, Cr, etc., high metal content=low pH) in the soil. Such toxicity, which may be as a result of excessive fertilization (Sun et al., 2016), affects not only plants, but also insects (Mogren and Trumble, 2010) and microorganisms, either beneficial or nonbeneficial. One such group of beneficial microorganisms is entomopathogenic nematodes (EPNs). The EPN infective juveniles (IJs) seek hosts in the soil and penetrate through natural openings, such as mouth, anus, or spiracles, to reach the hemocoel, where the symbiotic bacterial cells are released (Salvadori et al., 2012); both EPN genera can also penetrate through the cuticle of a suitable host. The IJs can survive for long periods in the soil without feeding, while remaining in a dormant state (Adams and Nguyen, 2002; Ehlers, 2001; Glazer, 1996; Womersley, 1990). The nematodes have a mutualistic association with *Xenorhabdus* and *Photorhabdus* bacterial species, for the Steinernematidae and Heterorhabditidae, respectively, and based on the insecticidal properties of this partnership they have been successfully exploited for biocontrol (Holajjer et al., 2014; Negrisoli et al., 2013; Stock and Blair, 2008; Shapiro-Ilan et al., 2011). EPNs occur naturally in the soil and their survival, host range, persistence, reproductive capacity, and infectivity can be affected by soil physio-chemical properties, temperature, soil organic matter, nutrient availability, and soil moisture (Kung et al., 1990b; Shapiro et al., 2000; Stock et al., 1999; Sun et al., 2016; Yadav, 2012), including and not limited to storage temperature (Ramakuwela et al., 2015; Strauch et al., 2000). The longevity of EPNs can also vary with varying controlled soil conditions (Shapiro-Ilan et al., 2006). However, general conclusions on nematode performance may not be uniformly agreed upon, as the effect of soil properties, such as soil pH and organic matter content, impact nematode species differently (Koppenhöfer and Fuzy, 2006). This highlights the importance of independently investigating EPN species across different soil properties, including pH.

Despite soil properties being one of the main factors that may hinder EPN potential, few studies have addressed the issue of differential tolerance to pH among EPN species and strains. As some EPNs persist in indigenous soils with pH ranges from 3.7 to 7 (Kanga et al., 2012) there are however, few studies that focus mainly on EPN performance across a wide range of the pH variable (San-Blas, 2013). The infectivity of some EPN populations can be affected when exposed to acidic soils, as the ability of the nematodes to find hosts can be inhibited in such soils (Fischer and Führer, 1990), while others tend to thrive in moderate to neutral pH conditions (Hussaini et al., 2004). Clearly, pH can hinder the efficacy of these nematodes, thereby affecting the intended level of biocontrol. Although pH has been shown to affect the survival of *Steinernema carpocapsae* and *Heterorhabditis indica* (Hussaini et al., 2004) and *S. carpocapsae* and *S. glaseri* (Kung et al., 1990b) differently, no studies have included a large representation of EPN populations across a wide pH range. Moreover, the effects of using different chemical compositions when measuring the impact of pH on organisms can vary (Burns, 1971), but has not been explored for EPNs. Of the widely investigated heterorhabditids and steinernematids, the former is more likely to be found in relatively higher soil pH, while the latter persists in lower soil pH (Rosa et al., 2000). Other than prevalence characterization and one study on progeny production post exposure (Hussaini et al., 2004), few studies have investigated the survival of EPNs after exposure to different pH conditions (Fischer and Führer, 1990; Hussaini et al., 2004; Kung et al., 1990a). As EPNs are applied to soil, which may have different pH levels at different layers, an improved understanding of their pH-sensitivity would be beneficial. The objective of this study was to investigate pH tolerance of 11 steinernematids and six heterorhabditids to different pH levels. Entomopathogenic nematode populations are expected to have varying tolerance to different pH levels. Furthermore, two chemicals were selected for preparation of pH solutions based on their differences in chemical reactions with water. Ammonium-acetate is hydroscopic in nature (Barthakur, 2007), this may lead to the depletion of oxygen contained in water. Citrate-phosphate has a tendency to prevent base hydrolysis, thus, the solution remains with an abundance of oxygen.

## Materials and methods

### Source of infective juveniles

Infective juveniles of 11 steinernematids and six heterorhabditids were sourced from the Fruit and Tree Nut Research Unit in the United States of America and the Agricultural Research Council-Small Grain Insect Pathology Laboratory in South Africa. This was done to promote the use of indigenous species in the two countries, especially in SA where regulations restrict introduction of exotic species (Table 1). The isolate SGI245 was newly identified as *Heterorhabditis bacteriophora* (nucleotide sequence accession number MW652709; 100% match on GeneBank). The isolate *H. bacteriophora* (HbHb) was originally isolated from Australia. However, the population may have adapted/evolved due to in vivo serial culturing in the laboratory (Grewal et al., 2002; Shapiro-Ilan et al., 2012). Infective juveniles of these populations were harvested from the final instar stage of the greater wax moth, *Galleria mellonella* (Linnaeus) (Lepidoptera: Pyralidae). This was achieved by infecting three larvae of *G. mellonella* with IJs of each population separately using the White trap method described by Kaya and Stock (1997). The collected IJs were stored in 600 ml flasks in sterile water at 10°C and were used within two weeks.

**Table 1.** List of entomopathogenic nematode populations that were tested for pH tolerance.

Isolate	Populations
S. biddulphi*	SGI246
S. beitlechemi*	SGI197
S. khoisanae*	R334
S. tophus*	R352
S. innovationi*	SGI60
Heterorhabditis bacteriophora*	SGI151; SASRI75; SGI245
*Steinernema carpocapsae^*	ScItalian; ScAll; ScCxrd
*S. glaseri^*	Sg4-8
*S. riobrave^*	Sr355
*S. feltiae^*	SfSN
*Heterorhabditis bacteriophora^*	HbHb; HbVS
*H. indica^*	HIHOM1

### Preparation of pH solutions

Two different acid-base chemical solutions were used to assess EPN tolerance to pH: 0.1 M acetic acid/ammonium hydroxide (ammonium-acetate, CH_3_COOH/NH_4_OH) and citric acid/disodium phosphate (citrate-phosphate, C_6_H_8_O_7_/Na_2_HPO_4_). A total of 10 pH solutions were prepared from each acid-base in 500 ml beakers and stored in 250 ml volumetric flasks. Working pH solutions, ranging from pH3 to pH10, were prepared from each of the bases. The primary solutions of pH2 and pH11 were prepared as follows: pH2 was prepared from the acid in combination with water (acetic acid: water and citric acid: water) and pH11 was each base with water (ammonium hydroxide: water and disodium phosphate: water). All solutions were adjusted with concentrated hydrochloric acid and 1 M sodium hydroxide as required. The pH was measured using a calibrated (buffer 11, 10, 7, and 4) Cyberscan pH 1100 pH/mV/°C/°F meter, with a silver–silver chloride ORP electrode (EUTECH Instruments, www.eutechinst.com).

### Experimental setup

The experimental design was a complete randomized design (CRD). From each of the 10 pH solutions, a 5 ml aliquot was pipetted into 50 ml centrifuge tube, using a 5 ml pipette. The viability of stored IJs was checked and the concentration of IJs for each population was adjusted to 1,200 IJs/ml. Aliquots of the suspension (5 × 10 µl) were pipetted onto a microscope glass slide to make droplets and the number of IJs per drop were counted under a dissecting microscope to obtain an average in *x*IJs/ml. The volume required for the desired concentration of 1,200 IJs/ml was calculated, as described by Kaya and Stock (1997). From the adjusted concentration, a 1 ml aliquot was transferred to an Eppendorf tube to settle the IJs. Excess water was pipetted out and the resulting pellet was transferred into the labeled centrifuge tubes and incubated horizontally for 24 hr at 25°C. This procedure was followed for all the populations. The experiment was repeated four times on different dates with a fresh batch of IJs.

### Infective juvenile survival assessment

After 24 hr incubation at 25°C, a 500 µl aliquot of the suspension was transferred into a 55 mm glass Petri dish with grids (improvising a nematode counting dish) and a total of 100 IJs were randomly examined under a dissection microscope and counted as dead or alive to calculate percentage survival (Kaya and Stock, 1997). To ensure that no live IJs were missed, non-moving juveniles were probed gently with a nylon brush bristle, and to avoid double counts, counts were performed from the top left to the right and bottom right to the left of the grids (Kaya and Stock, 1997).

### Statistical analysis

A factorial analysis of variance (ANOVA) was performed, comparing the two bases (ammonium-acetate and citrate-phosphate; with 10 pH levels per base) and 17 EPN isolates (6 heterorhabditids and 11 steinernematids). Thereafter, one-way ANOVA’s were performed to (i) compare survival among isolates at each pH and base; and (ii) compare survival of each isolate over the ten pH levels for each base. The standardized residuals showed an acceptable normal distribution (Shapiro–Wilks test; Shapiro and Wilk, 1965) after outliers were removed. The means were compared using Fisher’s unprotected *t*-test (least significant difference – LSD) at the 5% level of significance (Montgomery, 1984). All data analyses were performed using SAS statistical software 9.4 (SAS Institute Inc., 2016).

## Results

Although the three way interactions were highly significant (*p* < 0.001), the main results reflect on the one-way ANOVA’s.

### Survival of EPNs in ammonium-acetate pH solutions

**Figure 1:**
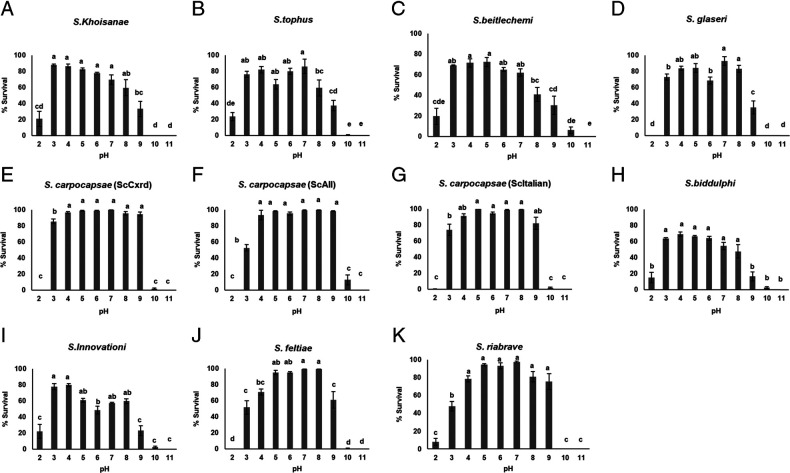
Survival of steinernematids infective juveniles in ammonium-acetate pH solutions 24 hr post incubation at 25°C. (A) *Steinernema khoisanae*, (B) *S. tophus*, (C) *S. beitlechemi*, (D) *S. glaseri*, (E) *S. carpocapsae* (ScCxrd), (F) *S. carpocapsae* (ScAll), (G) *S. carpocapsae* (ScItalian), (H) *S. biddulphi*, (I) *S. innovationi*, (J) *S. feltiae*, and (K) *S. riobrave*. Within each graph, bars (mean ± SE of individual observations) with different letters indicate significant differences at the 5% test level.

**Figure 2:**
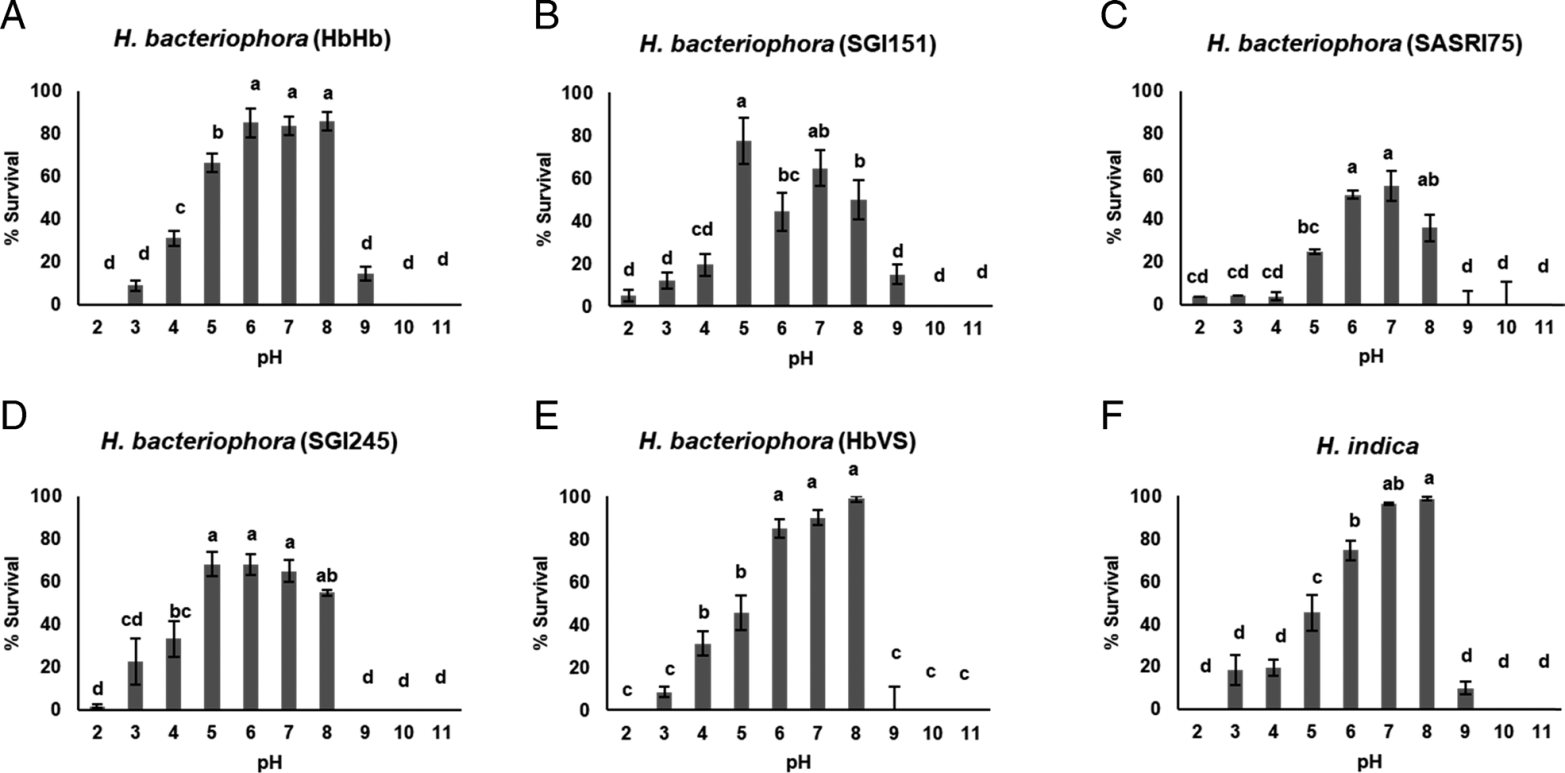
Survival of heterorhabditids infective juveniles in ammonium-acetate pH solutions 24 hr post incubation at 25°C. (A) *Heterorhabditis bacteriophora* (HbHb), (B) *H. bacteriophora* (SGI151), (C) *H. bacteriophora* (SASRI75), (D) *H. bacteriophora* (SGI245), (E) *H. bacteriophora* (HbVS), and (F) *H. indica*. Within each graph, bars (mean ± SE of individual observations) with different letters indicate significant differences at the 5% test level.

Poor survival at extreme pH levels (2, 10, and 11) was observed for all *Steinernema* populations (Fig. 1). None of the isolates survived at pH11, while survival at pH10 ranged from 0 to 12.75%. Likewise, survival at the extreme acidic level (pH2) ranged from 0 to 23.5% (Fig. 1). A notable improvement in survival across all isolates was at pH3 to pH9, with ScCxrd showing best mean survival of 96±5%. Survival among the heterorhabditids was generally lower compared to that among the steinernematids (Table 2), with only two isolates (*H. indica* and *H. bacteriophora* (HbVS)) showing >90% survival at any of the pH levels tested. The highest survival was noted in the range pH5 to pH8, with significantly lower survival at ≥pH9 for all heterorhabditid isolates tested. Likewise, there was low IJ survival toward the acidic extremes, pH2 to pH4, compared to the range pH5 to pH8 (Fig. 2). The three *S. carpocapsae* populations (ScCxrd, ScAll, and ScItalian) showed >80% survival over a wide pH range of 4 to 9 (Fig. 1). *Heterorhabditis indica*, *H. bacteriophora* (HbVS), *H. bacteriophora* (HbHb), *S. carpocapsae* (ScAll), and *S. feltiae* had the highest survival at pH8, while the rest of the populations started to display a decrease from this basic pH level. Only two populations, *S. carpocapsae* (ScCxrd) and *S. khoisanae*, showed good survival (>85%) at an acidic pH of 3, followed by *S. innovationi* and *S. tophus*. Notably, best survival of *S. khoisanae* was recorded in acidic solutions of pH3 to 5. Overall, *steinernema*tids consistently gave better survival in both acidic and alkaline solutions; *S. carpocapsae* (ScCxrd and ScItalian) was included in the highest ranking group for pH levels ranging from 3 to 9, and *S. carpocapsae* (ScAll) and *S. riobrave* were included in the highest ranking at pH ranges of 4 to 9 (Table 2). Among the *Heterorhabditis* spp., *H. bacteriophora* (HbHb and HbVS) and *H. indica* (HIHOM1), are the only species that consistently survived better at pH levels 6 to 8, apart from those that displayed broad tolerance of both acidic and alkaline pH environments (Table 2).

**Table 2.** Percentage survival of different entomopathogenic nematode populations at each of 10 pH levels post 24 hr exposure to ammonium-acetate or citrate-phosphate pH solutions.

Ammonium-acetate										
pH levels	2	3	4	5	6	7	8	9	10	11
*S. khoisanae* 334	20.75^a^	87.75^a^	86.50^abc^	82.50^abcd^	77.75^abc^	69.50^bcd^	59.50^bcde^	33.25^cdef^	0.00^b^	0.00^a^
*S. carpocapsae* (ScCxrd)	0.00^a^	86.33^a^	96.75^a^	98.75^ab^	99.00^a^	100^a^	95.50^a^	94.75^ab^	1.75^b^	0.00^a^
*S. beitlechemi* 197	19.50^a^	69.00^abc^	71.50^bc^	72.25^bcde^	65.00^cde^	62.00^d^	40.75^e^	30.25^cdef^	6.00^ab^	0.00^a^
*S. glaseri* SG4-8	0.00^a^	73.00^abc^	83.67^abc^	84.00^abcd^	68.33^cd^	92.67^a^	83.00^abc^	35.00^cde^	0.00^b^	0.00^a^
*S. tophus* 352	23.50^a^	76.25^abc^	81.75^abc^	63.75^de^	79.75^abc^	85.67^abc^	59.00^bcde^	37.25^cd^	0.00^b^	0.00^a^
*S. carpocapsae* (ScAll)	0.00^a^	52.50^bc^	93.25^a^	98.25^ab^	95.50^ab^	99.50^a^	99.75^a^	98.25^a^	12.75^a^	0.00^a^
*S. carpocapsae* (ScItalian)	0.25^a^	74.25^abc^	91.50^ab^	100^a^	94.75^a^	99.00^a^	99.50^a^	82.25^ab^	1.75^b^	0.00^a^
*S. biddulphi* 246	15.25^a^	63.50^abc^	69.25^c^	66.25^de^	64.00^cde^	54.25^d^	47.75^de^	16.50^def^	2.50^ab^	0.00^a^
*S. innovationi* 160	22.25^a^	78.00^ab^	79.75^abc^	61.00^de^	48.75^de^	57.50^d^	59.75^bcde^	23.00^def^	2.50^ab^	0.00^a^
*S. feltiae* FSN	0.00^a^	51.75^bc^	70.75^bc^	95.25^abc^	95.25^ab^	99.50^a^	99.75^a^	61.25^bc^	0.75^b^	0.00^a^
*S. riobrave* 355	8.00^a^	48.00^cd^	78.50^abc^	94.25^abc^	93.00^ab^	97.50^a^	80.75^abcd^	75.50^ab^	0.00^b^	0.00^a^
*H. bacteriophora* (HbHb)	0.00^a^	9.00^e^	31.25^d^	66.50^de^	85.25^abc^	84.00^abc^	86.00^ab^	14.50^def^	0.00^b^	0.00^a^
*H. indica* HIHOM1	0.00^a^	18.50^e^	19.75^de^	45.50^ef^	74.50^bc^	96.75^a^	98.75^a^	10.00^def^	0.00^b^	0.00^a^
*H. bacteriophora* (SASRI75)	3.75^a^	4.00^e^	3.75^e^	24.50^f^	51.25^de^	55.25^d^	35.75^e^	0.00^f^	0.00^b^	0.00^a^
*H. bacteriophora* (SGI151)	5.00^a^	12.25^e^	19.50^de^	77.50^abcd^	44.25^e^	64.75^cd^	49.75^cde^	15.00^def^	0.00^b^	0.00^a^
*H. bacteriophora* (SGI245)	1.67^a^	22.67^de^	33.33^d^	68.33^cde^	68.00^cd^	65.00^cd^	55.00^bcde^	0.00^f^	0.00^b^	0.00^a^
*H. bacteriophora* (HbVS)	0.00^a^	8.50^e^	31.25^d^	45.75^ef^	85.00^abc^	90.25^ab^	99.00^a^	0.67^ef^	0.00^b^	0.00^a^
LSD (0.05)	25.85	29.00	20.97	26.97	22.49	21.58	35.03	34.67	10.37	0.00
Citrate-phosphate										
pH levels	2	3	4	5	6	7	8	9	10	11
*S. khoisanae* 334	0.00^e^	63.25^e^	64.00^d^	67.75^e^	76.75^fg^	78.00^f^	81.50^def^	81.50^cd^	71.75^cde^	18.75^ef^
*S. carpocapsae* (ScCxrd)	85.75^a^	93.25^ab^	100^a^	100^a^	99.75^a^	99.75^a^	100^a^	100^a^	97.75^ab^	100^a^
*S. beitlechemi* 197	2.75^e^	69.25^de^	73.25^cd^	75.25^de^	79.50^efg^	80.25^ef^	86.50^bcdef^	82.75^bcd^	76.25^bcde^	24.00^def^
*S. glaseri* SG4-8	27.75^de^	93.00^ab^	97.75^ab^	88.50^abcd^	94.50^abc^	98.00^ab^	93.00^abcd^	95.50^ab^	78.50^abcd^	7.25^f^
*S. tophus* 352	1.25^e^	82.50^abcd^	83.50^abcd^	87.75^abcd^	90.00^abcde^	91.50^abcde^	95.50^ab^	93.50^abc^	92.50^abc^	23.00^def^
*S. carpocapsae* (ScAll)	73.00^ab^	100^a^	99.75^a^	99.75^a^	100^a^	98.50^ab^	100^a^	99.75^a^	99.50^a^	100^a^
*S. carpocapsae* (ScItalian)	69.50^ab^	97.25^ab^	99.25^ab^	95.00^abc^	100^a^	99.75^a^	97.00^ab^	99.75^a^	100^a^	98.50^a^
*S. biddulphi* 246	1.50^e^	73.75^cde^	71.00^cd^	83.75^bcd^	92.75^abcd^	85.50^cdef^	91.25^abcde^	90.25^abc^	75.75^bcde^	48.75^cde^
*S. innovationi* 160	0.00^e^	73.00^cde^	83.50^abcd^	68.00^e^	71.75^g^	85.75^cdef^	82.25^cdef^	87.00^abc^	86.25^abc^	45.50^cde^
*S. feltiae* FSN	0.00^e^	85.00^abcd^	95.50^ab^	92.50^abc^	96.25^ab^	97.25^abc^	99.25^ab^	98.00^a^	96.25^ab^	88.00^ab^
*S. riobrave* 355	33.25^cd^	99.25^a^	100^a^	99.00^a^	99.50^a^	100^a^	96.50^ab^	99.50^a^	99.75^a^	60.00^bc^
*H. bacteriophora* (HbHb)	61.00^abc^	91.00^abc^	96.25^ab^	93.75^abc^	94.75^abc^	94.75^abcd^	94.50^abc^	93.50^abc^	55.25^e^	0.00^f^
*H. indica* HIHOM1	46.50^abc^	98.25^ab^	97.25^ab^	99.25^a^	97.50^ab^	99.50^a^	98.00^ab^	95.25^ab^	71.00^cde^	0.67^f^
*H. bacteriophora* (SASRI75)	66.50^ab^	80.00^bcde^	78.00^bcd^	83.75^bcd^	87.00^bcdef^	84.00^def^	73.75^f^	73.00^d^	77.25^bcde^	54.50^cd^
*H. bacteriophora* (SGI151)	64.25^ab^	85.00^abcd^	88.50^abc^	90.00^abcd^	82.25^defg^	86.75^bcdef^	87.50^abcde^	82.50^bcd^	82.75^abcd^	30.00^cdef^
*H. bacteriophora*. (SGI245)	77.75^a^	84.75^abcd^	88.25^abc^	81.50^cde^	82.75^cdefg^	81.75^ef^	78.50^ef^	83.25^bcd^	84.75^abcd^	42.50^cde^
*H. bacteriophora* (HbVS)	34.00^cd^	96.50^ab^	99.50^a^	98.25^ab^	98.00^ab^	97.50^abc^	97.75^ab^	98.00^a^	64.00^de^	1.00^f^
LSD (0.05)	30.13	18.76	21.27	15.09	12.04	12.13	12.76	13.42	22.19	32.11

**Note:** For a given base, means within a column followed by different letters differed significantly at the 5% test level.

### Survival of EPNs in citrate-phosphate pH solutions

**Figure 3:**
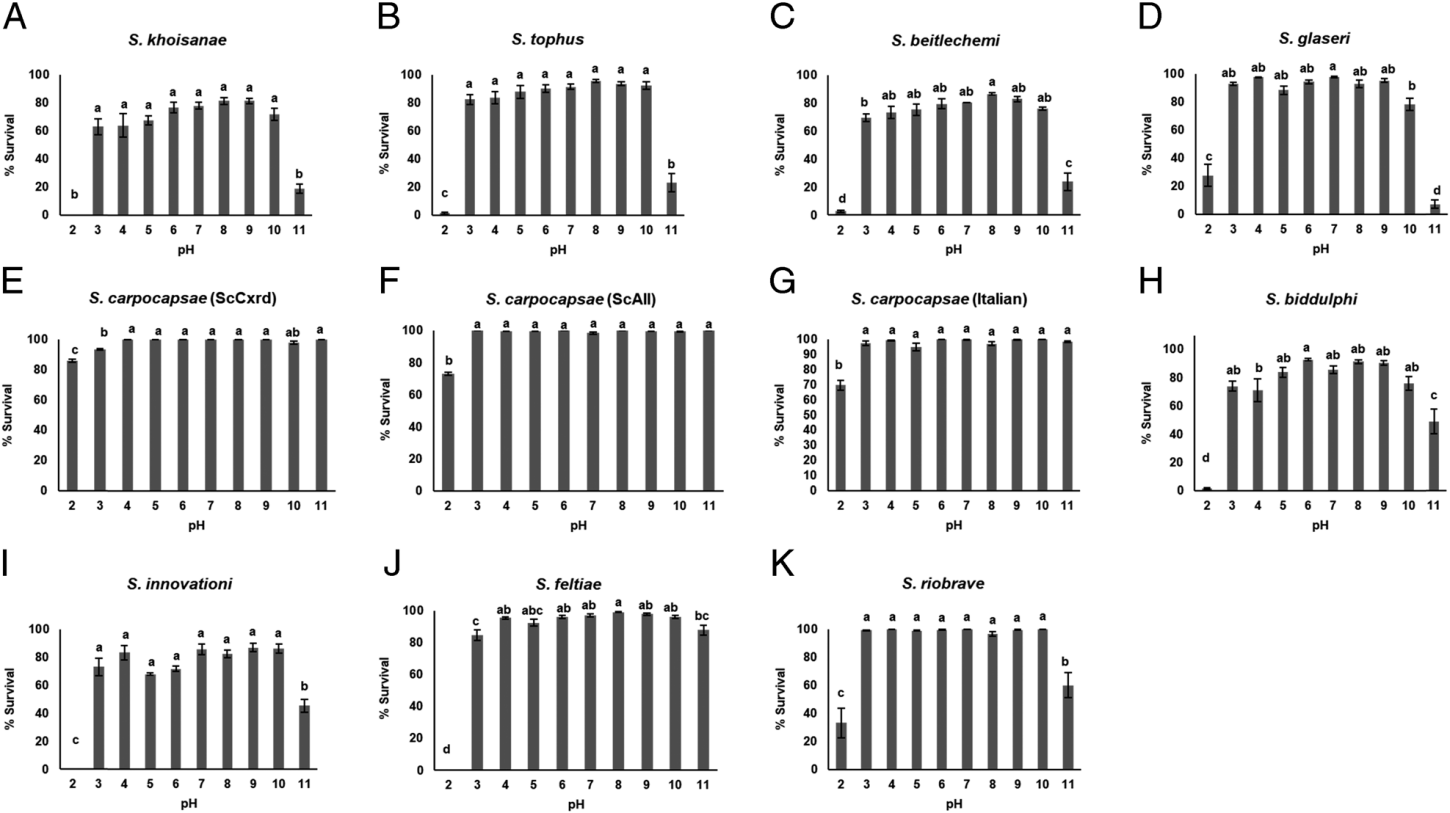
Survival of steinernematids infective juveniles in citrate-phosphate pH solutions 24 hr post incubation at 25°C. (A) *Steinernema khoisanae*, (B) *S. tophus*, (C) *S. beitlechemi*, (D) *S. glaseri*, (E) *S. carpocapsae* (ScCxrd), (F) *S. carpocapsae* (ScAll), (G) *S. carpocapsae* (ScItalian), (H) *S. biddulphi*, (I) *S. innovationi*, (J) *S. feltiae*, and (K) *S. riobrave*. Within each graph, bars (mean ± SE of individual observations) with different letters indicate significant differences at the 5% test level.

**Figure 4:**
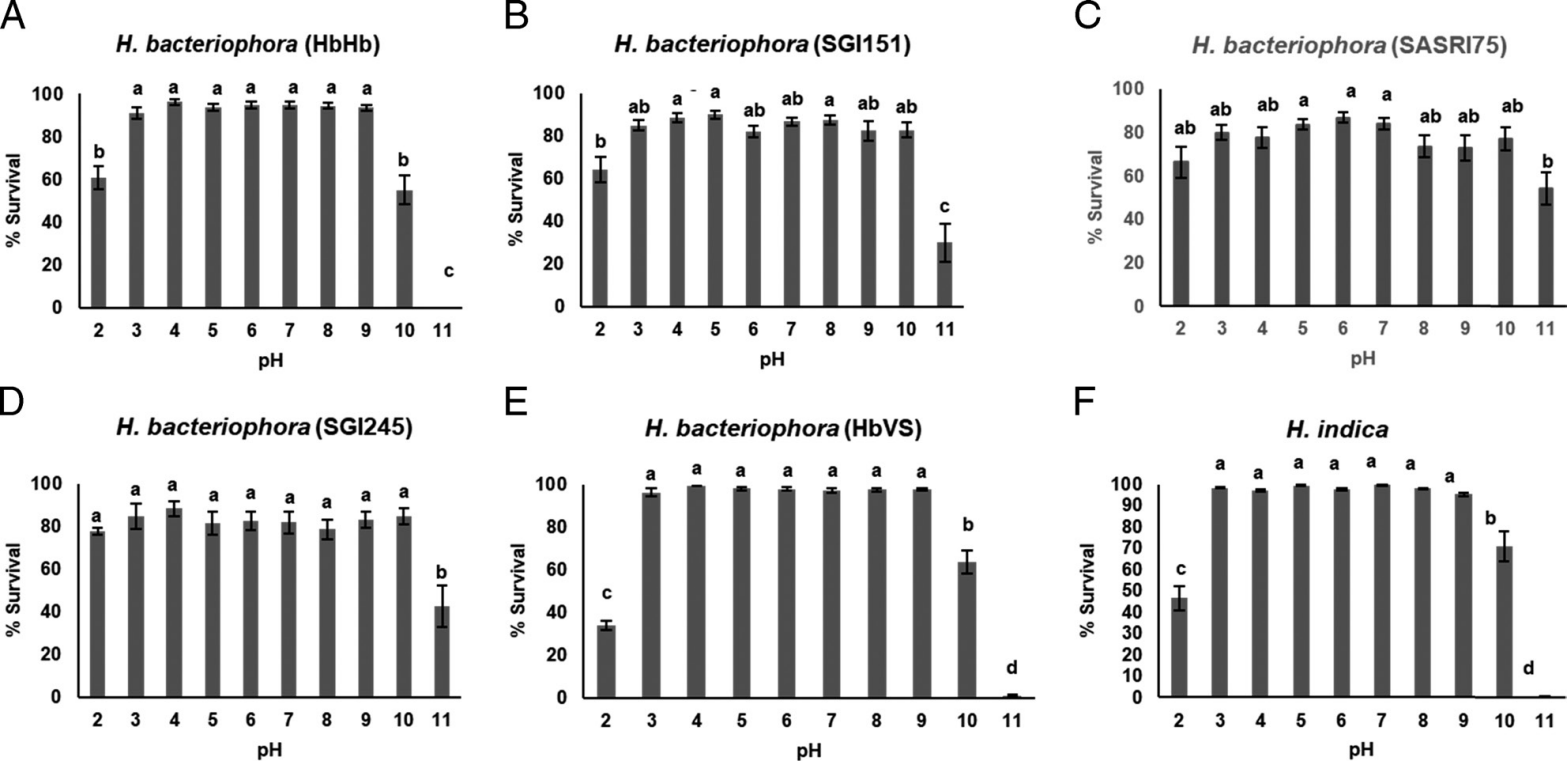
Survival of heterorhabditids infective juveniles in citrate-phosphate pH solutions post incubation at 25°C. (A) *Heterorhabditis bacteriophora* (HbHb), (B) *H. bacteriophora* (SGI151), (C) *H. bacteriophora* (SASRI75), (D) *H. bacteriophora* (SGI245), (E) *H. bacteriophora* (HbVS), and (F) *H. indica*. Within each graph, bars (mean ± SE of individual observations) with different letters indicate significant differences at the 5% test level.

Almost 80% IJ survival was evident in citrate-phosphate (mean 79.3%; all pH levels pooled) compared to only 46% in ammonium-acetate. The lower level of discriminating power yielded no significant differences among pH levels 4 to 9 within any of the steinernematids (Fig. 3). In an extreme alkaline environment (pH11), lowest survival was 7% for *S. glaseri* and not statistically different from three other steinernematids, *S. khoisanae*, *S. beitlechemi*, and *S. tophus* (Table 2). At this pH, four populations (*S. feltiae, S. carpocapsae* (ScItalian), *S. carpocapsae* (ScCxrd), and *S. carpocapsae* (ScAll)), showed ≥88% survival and not statistically different from one another (Table 2). At pH2, survival of the three top performers (*S. carpocapsae* populations) ranged from 70 to 86%. No significant differences were detected in the pH range 3 to 9 within any of the six heterorhabditids tested (Fig. 4). Highest survival at the extremes, pH2 and pH11, was 78% (*Heterorhabditis bacteriophora* (SGI245)) and 55% (*H. bacteriophora* (SASRI75)), respectively (Table 2).

## Discussion

Survival of non-feeding IJs can be affected by various abiotic factors, including the pH of the soil, therefore, the survival mechanism of EPNs has a pivotal role in their ability to persist in soil conditions (Glazer, 1996). This is the first study to investigate pH tolerance of a variety of EPNs to a wide pH range. Entomopathogenic nematode species showed a variation in pH tolerance, with an apparent reduced survival of IJs observed beyond neutral pH in ammonium-acetate solution in the current study. This could be due to the fact that nematode performance may not be generalized, as the effect of soil properties, such as soil pH and organic matter content, impact nematode species differently (Koppenhöfer and Fuzy, 2006). EPN species or even strains have been observed to have diverse temperature optima (Grewal et al., 1994, 1996; Mason and Hominick, 1995). Similarly to temperature, the difference in pH tolerance may be attributed to species differences. Our findings are similar to an observation recorded by Salamún et al. (2014), where nematode communities collapsed at increased soil pH levels. The heterorhabditids displayed the lowest survival in the acidic pH levels in ammonium-acetate, which is consistent with results recorded by Hussaini et al. (2004). Strauch et al. (2000) observed that *H. indica* and all the *H. bacteriophora* only showed improved survival at pH4 and pH5 in Ringer’s solution with the pH adjusted using NaOH and phosphoric acid (H_3_PO_4_), contrary to the current study where peak survival was observed from pH5 to 8 for these species in ammonium-acetate (adjusted with NaOH and HCl).

Fischer and Führer (1990) reported that activity of *Steinernema kraussei* (Steiner, 1923) Travassos, 1927 was low at pH levels 3.2 to 3.5, while using citrate-phosphate to manipulate soil pH levels. In contrast to Fischer and Führer (1990), our results indicated >63% survival in citrate-phosphate for both steinernematids and heterorhabditids at pH3 and pH4. The difference may be due to other soil parameters in the two studies that may interact with various nematode species. As Koppenhöfer and Fuzy (2006) indicate, soil type effects cannot be generalized to all EPNs as different soil properties within various soil types affect EPN species differently.

All nematodes displayed exceptional survival in pH ranges 3 to 10 and a survival decline in selected EPNs at pH11 and pH 2 in citrate-phosphate. This observation is contrary to Fischer and Führer (1990), who used citrate-phosphate and found increased nematode activity from pH4. The *S. carpocapsae* juveniles of all populations tested (ScCxrd, ScAll, and ScItalian) had a high survival range (>50%) at pH3 to 9 in ammonium-acetate; similar results were observed by Hussaini et al. (2004) and Strauch et al. (2000). However, the result was contrary to Kung et al. (1990a) when citrate-phosphate was used, as EPN survival increased with increasing pH levels. *Steinernema tophus* and *S. glaseri* displayed a ‘mirror like’ survival behavior to each other, which may be because *S. tophus* is morphologically classified under the *glaseri* group (Çimen et al., 2014). From the literature, it is evident that some species are affected by acidic soils, and others by alkaline soils, yet there are cases where some populations survive in both acidic and basic soils (Dzięgielewska and Skwiercz, 2018). This is corroborated by the current study where several species (all *S. carpocapsae* populations and *S. riobrave*) survived in both extreme acidic and alkaline solutions for ammonium-acetate.

Nematodes are believed to be adapted to the soil ecological conditions found in the region they were isolated (Kung et al., 1991; Molyneux, 1985). Thus, we expect that pH tolerance may be tied to the region of geographic isolation, and our results support this premise though the study focused on the original place of isolation and the isolated species were not tested at different geographic regions. For example, in the current study, *S. khoisanae* showed better survival in the acidic region of the ammonium-acetate pH solutions which may be linked to its isolation from acidic soils with average pH of 3.9 (Hatting et al., 2009). Populations of *S. beitlechemi* and *S. biddulphi,* isolated from the Eastern Free State area of South Africa (Cimen et al., 2016a, b), where average soil pH levels tend to be 4.51 (Sosibo et al., 2017), showed >63% survival at pH3 to pH5 for both solutions. In corroboration to Kung et al. (1990a), they showed decreased survival beyond the neutral pH in ammonium-acetate. At pH3 to pH6, *S. tophus* survived >60 and >80% in ammonium-acetate and citrate-phosphate, respectively. *Steinernema tophus* was isolated from a vineyard (Hatting et al., 2009), where pH levels tend to range from 4 to 6 (Bargmann, 2003). This finding agrees with the observed steinernematids’ increased survival at acidic pH levels as compared to the heterorhabditids where survival only picks up from pH5 to pH6 in ammonium-acetate in the current study. On the other hand, it was interesting to note that populations that have broad tolerance to pH [*S. carpocapsae* (Sc-All, Cxrd, and Italian) and *S. riobrave*] are not specialized to one extreme pH or another and their tolerance is not only tied to the pH of their origin. On a positive note, given that EPNs appear adapted to their soil environment of origin, using indigenous EPNs should therefore negate the need to introduce foreign populations (Gungor et al., 2006). This is also true considering that pH is not the only parameter that should be considered when choosing EPNs for biocontrol purposes. It is worthwhile to consider that indigenous EPNs are already adapted to their local habitat and to the target pests present in a specific area.

Some generalizations can be drawn from our results in terms of differences among the genera and between the two solutions used. Generally, the steinernematids survived at a wider pH range than the heterorhabditids that survived better from neutral to slightly alkaline pH (7-8) in ammonium-acetate solutions. This is contrary to Li et al. (2019) results, where they observed that *Heterorhabditis megidis* and *H. bacteriophora* (HBN, NJ, CD-11, and NT-82), preferred an acidic pH range of 4.32 to 5.04. Looking at the pH preference of the three *steinernema* species (*S. carpocapsae* Sc-All, *S. felitae* Sf-SN, and *S. riobrave*) that were common in this study and that of Li et al. (2019), they showed wider pH tolerance with no significant differences at pH 4-9 (*S. carpocapsae* Sc-All and *S. riobrave*) and 5-8 for *S. felitae* Sf-SN when ammonium-acetate was used. In Li et al. (2019) study, they showed narrow pH preference of 5.78-6.57, 5.58-6.95, and 5.76-6.62 for *S. carpocapsae* Sc-All, *S. felitae* Sf-SN, and *S. riobrave*, respectively. In the current study, the extremely acidic and extremely alkaline pH levels had a deleterious effect on all populations, especially the heterorhabditids. The decreased survival of EPNs at pH2 and pH11 could be attributed to the solutions consisting of almost purely the acid or base, respectively. Additionally, all EPNs exhibited higher survival in citrate-phosphate solution than in ammonium-acetate, and only pH2 and pH11 were unfavorable to specific populations. As acidic solutions containing ammonium tend to repel nematodes (Pye and Burman, 1981), low EPN survival in ammonium-acetate may be attributed to the hydroscopic nature of the ammonium-acetate (Barthakur, 2007), leading to the depletion of oxygen (contained in water), which the nematodes needed for survival, resulting in high IJ mortality. In contrast, citrate-phosphate has a tendency to prevent base hydrolysis, where the use of water would break molecules apart; thus, an abundance of oxygen for the IJs may result and thereby explain the enhanced survival in this solution relative to ammonium-acetate. The ammonium-acetate solutions were more discriminative of the EPN species survival at different pH levels. The citrate-phosphate had better IJ survival in a wider pH spectrum, with *H. bacteriophora* (SASRI75) displaying survival above 50% unhindered by the differences in pH levels. Consequently, it would be advisable to conduct such experiments using citrate-phosphate for the benefit of oxygen availability for the nematodes. However taking into account the ability to distinguish the tolerance of the EPNs, ammonium-acetate displays the properties better as opposed to citrate-phosphate and it is a common extraction agent used in soil nutrient testing laboratories (Saarela, 2002). On that note, our recommendations for application of different EPNs at different pH ranges were based on survival in ammonium-acetate buffer. Additionally, the optimum pH level for plant production was considered.

Entomopathogenic nematode survival was affected by the varying pH levels. These findings can be of use when selecting EPNs for biological control purposes. The four *Steinernema* spp., *S. carpocapsae* (ScCxrd, ScAll, and ScItalian) and *S. riobrave* showed consistently higher survival in both acidic and alkaline solutions, suggesting that they may be applied across the board in both acidic and alkaline soils. Notably, all South African EPNs showed peak survival at ≤pH7 in the more discriminative ammonium-acetate base. Specifically, four steinernematids (*S. khoisanae, S. beitlechemi, S. biddulphi* and *S. innovationi*) showed superior adaption to an acidic environment. Application of these species in South Africa, where low-pH soils are commonly associated with major crop commodities (Hatting and Malan, 2017), especially in provinces like KwaZulu-Natal, Mpumalanga and Western Cape (ARC–ISCW, 2005), will avoid dealing with regulations which restricts importing exotic EPNs. It was discovered that chemicals used for the manipulation of the pH affect their survival. However, it is important that they have to be discriminative of the species behavior post exposure. Nevertheless, it is speculated that IJ survival at different pH levels does not necessarily predict their virulence. There is scant information on the effect of pH on EPNs’ ability to infect hosts and reproduce post exposure. Therefore, it would be interesting for future studies to focus on infectivity and progeny production of the surviving IJs post exposure to different pH levels. Nonetheless, our results provide an initial baseline to build on.
